# Review of biomedical signal and image processing

**DOI:** 10.1186/1475-925X-12-88

**Published:** 2013-09-08

**Authors:** Edward J Ciaccio

**Affiliations:** 1 Department of Medicine – Division of Cardiology, Columbia University Medical Center, 630 West 168th Street, New York, NY 10032, USA

**Keywords:** Biomedical, Fourier, Image processing, Signal processing, Textbook

## Abstract

This article is a review of the book “Biomedical Signal and Image Processing” by Kayvan Najarian and Robert Splinter, which is published by CRC Press, Taylor & Francis Group. It will evaluate the contents of the book and discuss its suitability as a textbook, while mentioning highlights of the book, and providing comparison with other textbooks.

## Book details

Biomedical Signal and Image Processing, second edition, Review of Biomedical Signal and Image Processing, CRC Press, Taylor & Francis Group, Boca Raton, Review by Edward J. Ciaccio, PhD, Columbia University in New York by Kayvan Najarian and Robert Splinter; 2012: 411 pages, List Price. $99.95, ISBN number: 9781439870334, Ebook ISBN 9781466506558.

## Introduction

The book ‘Biomedical Signal and Image Processing’ by Kayvan Najarian and Robert Splinter is published in hardcover and electronic form by CRC Press, a company well-known for scientific textbooks. It will be of interest to biomedical engineers and scientists who need to process data, and is meant to be used as a textbook for undergraduate course-level material and also as a reference text. In reviewing the book, I will attempt to use the perspective of a student, and to ask questions and critique the text as a typical student at the upper undergraduate level would be likely to do. The text is generally light on equations and heavy on concepts. This can be very helpful to the student new to the field if the conceptual information is provided with thoroughness and clarity. My thoughts on the book are presented in sequence from first chapter to last. I have tried to state clearly the strong points as well as any noticeable deficiencies.

### Critique of the formatting of book and text

I read Biomedical Signal and Image Processing in its electronic form on my laptop computer, which has a 12.1 inch screen and a resolution of 1280 × 800 pixels. I believe that the print form of the book comes with Matlab programs. These programs may have been included with the electronic form, but they were not evident to me. On the publisher’s website (crcpress.com) the reader can download auxiliary materials (figures and tables) after a search for the book’s webpage.

The software provided by the publisher to read the book electronically is entitled ‘VitalSource Bookshelf’.

First I would like to comment on the correctness of the text. It is disappointing that there are a substantial number of typos and grammatical errors even though this is the second edition of the book. Another problem, or at least an annoyance, concerning the electronic form is that when you highlight some text and copy, it also copies the book reference information. This reference information will need to be removed each time you paste if you are trying to make some notes from the electronic book text.

**Figure 1:**
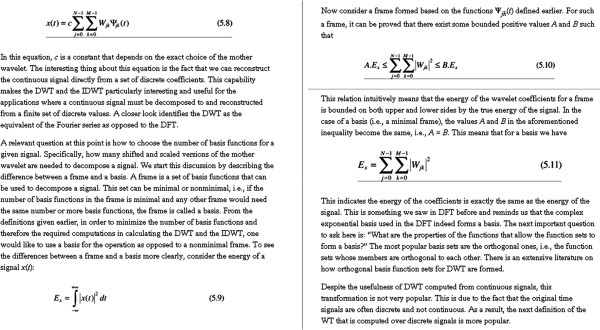
Legend - equations from the wavelets analysis chapter of the book.

In terms of the visual aspects of the electronic form, some of the equations in the text are images, while others are actual text. Another difficulty is that the numbered equations, separate from the text, are all images and they are a bit blurry. My laptop has the ability to make much sharper images when they are available. Examples of what I feel are blurred equations can be observed in Figure 1. The tinier symbols in the numbered equations are a bit blurred. For ease of reading, these could be sharper.

It is possible to add reader comments to the electronic form of the book, and to highlight the text in color as one would do with a highlight pen. This is very useful. A concern is that it is not clear when the highlights and comments are saved. There does not seem to be a way to save the result until one exits the software program.

## Content of the book

One would expect that the material covered in Biomedical Signal and Image Processing would primarily be about biomedical signal and image processing, the title of the book. However, it is spotty in its comprehensiveness and there are entire chapters devoted to peripheral topics. There are no citations given for any of the material and no reference texts are shown.

In terms of comprehensiveness, the first chapter, Introduction to Digital Signal and Image Processing is helpful to the discussion. Basic introductory materials about signal and image processing are provided. In the second chapter however, which is devoted to the Fourier Transform, a very important concept in biomedical engineering, details are lacking. The reader is provided the definition of the Fourier transform. The following equation is provided:

(2.1) $$G\left(f\right)=\int_{-\infty }^{+\infty }e^{-j2\mathrm{\pi ft}}\mathrm{dt}$$

The frequency variable *f*, imaginary number *j*, and continuous signal *g(t)* are defined in the text. However other variables lack explanation. What is the purpose of the exponential function in the transformation equation, for example? What is the purpose of *2π* and *t*? How is the exponential related to the sine waves that are used for signal transformation? How does it work to convert a signal to basis functions and power spectrum? Instead of explaining these concepts, the authors devote a large portion of the chapter to transforming simple mathematical functions.

The two-dimensional discrete Fourier transform is also described briefly in this chapter. What I would like to have seen is a description of the relationship between biomedical image and the two-dimensional Fourier transform that results. It would have been interesting to see images with visibly periodic patterns and their Fourier representation, then work up to real biological images which may have periodic components, and show their transformation. This is done for example, in a popular bioimaging text [1].

I expected toward the end of the chapter to see a section on the Fast Fourier Transform and the Cooley-Tukey algorithm, but no such information is provided on this topic. Knowledge of the method would have been helpful toward understanding the mathematics of Fourier analysis. It is also a question that appears on many graduate level examinations including PhD qualifiers.

The book then shifts to image processing techniques, which are covered briefly in Chapter 3, entitled Image Filtering, Enhancement, and Restoration, and in Chapter 4, Edge Detection and Segmentation of Images. The basic aspects of contrast enhancement, histogram equalization, masking, low and high pass filtering, edge detection, and image segmentation are provided. These two chapters provide most of the image processing information that is covered in the book. Yet most image processing texts are much larger and more comprehensive [1]. These two chapters can be used as a basic introduction to image processing only.

Very important to biomedical engineering is the topic of wavelets, which is considered in chapter 5. The authors lead into the concept by describing the short-time Fourier transform, which is helpful and clear. They show how windowing at short intervals is useful to capture time-variant information, and how the analysis window size is very important for interpreting time-variant events. There are suitable illustrations for this purpose. The concept of using analysis windows other than the rectangular type is also introduced. The authors mention triangular and trapezoidal analysis windows, and that more weight would be assigned to the central part of the window when these shapes are used. However this is the only place in the book where these windows are mentioned at all, and they are not described. The concept of weighting and how window type affects the analyzed signal should be detailed.

The actual description of wavelets is very good in my opinion. The authors nicely describe the relationship of scale and shift for wavelet transformation, and how this relates to the short-time Fourier transform. However I would have preferred to see more information about the mother wavelet and what it does. Why exactly are there different mother wavelets that are used for transformation? How does the choice of mother wavelet affect the result? If one chooses one particular mother wavelet over another, can it significantly affect the time-frequency information that is obtained? Can it affect the conclusions that are reached by implementing wavelet analysis for biomedical application? If so, why then are wavelets useful when the results and conclusions depend upon the choice of mother wavelet? If the results and conclusions are robust to mother wavelet choice, why and how? These are the kind of need-to-know topics that would be very helpful to present in any text that is more conceptually oriented and geared toward students.

The authors also mention in the chapter that a continuous time series signal can be reconstructed from discrete wavelet coefficients. How can this be? Is it because the mother wavelet itself is a continuous function? It would be helpful to see an example of how this works.

The authors then explain the difference between a frame and a basis. They state that it can be proven that there exists bounded positive values A and B such that Eq. 5.10 is valid (see Figure 1). Note this equation is blurry. But more importantly, what are A.E_x_ and B.E_x_? As a reader, I would like to know. What does this notation mean? Is it a mathematical shorthand or a typo? Particularly for undergraduate level students, this should be explained.

**Figure 2:**
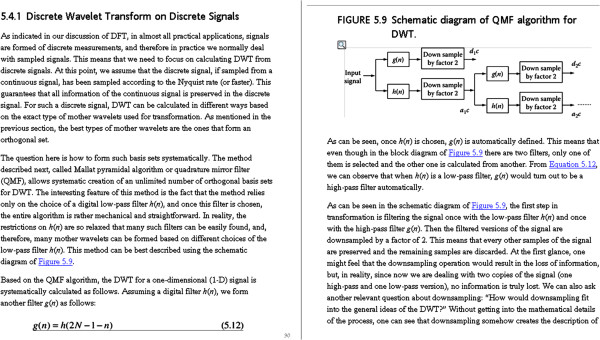
Legend - diagram of the Quadrature Mirror Filter from the book.

A major part of the wavelets chapter is devoted to the Quadrature Mirror Filter (QMF) algorithm, which in many cases is used to implement wavelet analysis. The authors’ diagram for QMF is shown in Figure 2. It is unclear from the text how and why this implementation can replace discreet shift and scale of the mother wavelet at multiple levels for wavelet analysis. The diagram in the book, reproduced in Figure 2, shows that the input signal is filtered by the QMF algorithm separately for both high and low pass content. Why? How do the high and low pass filters relate to shift and scale? We are told that the high and low pass form of the digital filter can be obtained from Equation 5.12, reproduced below:

(5.12) $$g\left(n\right)=\left(2N-1-n\right)$$

but are not told what the variables n and N represent. It is important to define all variables as they are used in equations. Concerning the QMF algorithm diagram (Figure 2), the authors say:

‘As can be seen, once h(n) is chosen, g(n) is automatically defined. This means that even though in the block diagram of Figure 5.9 [shown in Figure 2 in this review] there are two filters, only one of them is selected and the other one is calculated from another.’

This statement is unclear to me. What is calculated from what? More detail is needed. The authors continue:

‘As evident from Figure 5.9 [shown in Figure 2 in this review], the signal transformation and decomposition can be repeated for as many levels as desired. If one wishes to terminate the operation at the first level, two coefficients are found, d1c (the high-pass coefficient on the first level) and a1c (the low-pass coefficient on the first level). … This would result in two new coefficients: d2c (the low-pass coefficient on the second level) and a2c (the high-pass coefficient on the second level).’

I am not sure if I understand this passage. If d1c is the high-pass coefficient on the first level, why is a2c the high-pass coefficient on the second level? Shouldn’t it be d2c? If not, the authors need to explain more clearly in the text why the variable associated with each type of filter changes letter. Similarly, if a1c is the low-pass coefficient on the first level, why is d2c the low-pass coefficient on the second level? The authors should explain how the coefficients work, and which of g(n) and h(n) is the high pass and low pass filter at each level.

The text then continues:

‘From the preceding equations, one can see that the mother wavelet of the operation is uniquely identified and represented by the filter h(n) or, in other words, the role of the mother wavelet is somehow replaced by h(n).’

Please explain how this works. The concept should not have to be understood just from the equations, but through the text as well.

The following chapter, Chapter 6, entitled ‘Other Signal and Image Processing Methods’ was of interest to me because I thought that some of the major additional methods used in signal and image processing would be discussed, such as eigenanalysis, linear predictive methods, and adaptive filtering. However they were not included. The authors devote a page to fractal dimension, which is helpful, but other aspects of nonlinear analysis and chaos theory are not presented – perhaps an entire chapter or more could be devoted to this field, which is quite important to signal and image processing. The cosine transform is then discussed. Here is another example of a transform, like the Fourier transform, for which it would be very useful to know the relationship of the equation to its sinusoidal basis. How is the cosine transform similar to or different from the Fourier transform? It would be an important question for me as a student. Why is only the cosine wave used for transformation? Is there a sine transform?

Several chapters are then devoted to peripheral topics. Chapter 7 is devoted to clustering and classification. Although pattern recognition may or may not be considered to be a branch of signal and image processing, it is sufficiently related that I agree with the authors that it merits a chapter. Topics such as k-means and the maximum likelihood methods are discussed. Part II of the book however, is mostly devoted to the physiologic aspects of biomedical signals. Although its title is ‘Processing of Biomedical Signals’ it really is short on processing and long on physiology. These chapters consist of Chapters 8–12, which are entitled, respectively, ‘Electrical Activities of the Cell’, ‘Electrocardiogram’, ‘Electroencephalogram’, ‘Electromyogram’, and ‘Other Biomedical Signals’. The authors concentrate on bioelectric signals, mostly to the exclusion of biomechanical, biomagnetic, and bioacoustic signals. Only Chapter 12, ‘Other Biomedical Signals’, provides brief descriptions of selected non-electrophysiologic signals – the blood pressure signal, magnetoencephalogram, and respiratory signals.

Part III of the book is entitled ‘Processing of Biomedical Images’. Similar to Part II, there is less description of processing methods, and more description of biomedical image formation. In this Part, the concepts behind the source of the image are discussed in great detail, in Chapters 14 – 18, which are respectively, Principles of Computed Tomography, X-Ray Imaging and Computed Tomography, Magnetic Resonance Imaging, Ultrasound Imaging, Positron Emission Tomography, and Other Biomedical Imaging Techniques. Though interesting, it would be helpful to learn more about actual image processing techniques, of which there are devoted many large textbooks and reference manuals.

## Conclusions

Overall I would give the book a grade of C-. The student exposed to the material for the first time would learn some helpful concepts, but would not likely understand all concepts. The text offers little in the way of ‘value added’ i.e. understanding of larger issues related to biomedical signal and image processing. Many signal and image processing techniques are not even covered. There would be gaps in information which would necessitate the use of a supplementary text to fill in the blanks. I think, the book might be helpful as an accessory text for its coverage in more detail of the physiologic origin of certain signals and images. I would not use this as a sole textbook or reference book, since details are lacking, and presence of typos and grammatical mistakes, as well as blurred equations, can result in a lack of clarity. The cost of the book is more than I would pay for its helpfulness – in my opinion it should be discounted. Otherwise, CRC Press has an obligation to improve the editing and proofreading to maintain high quality and standard. Reviews of the 1st edition were not so good; the publishing house should have corrected the errors and improved the text.

My personal choices for reference texts in this field are noted below [1,2]. These texts are understandable and fairly comprehensive, and I have used them as course textbooks. The signal processing text that I refer to may be out of print [2], but still appears to be available in electronic format. These texts have few typos and grammatical errors. They have working and practical examples. They explain the concepts well. These are the kind of books which would be assistive in the well-rounded education of the engineering student, enabling the ready application of the concepts in their future work.

## Competing interests

The author has no competing interests and no disclosures to report.
